# Laboratory Diagnosis of Bovine Abortions Caused by Non-Maintenance Pathogenic *Leptospira* spp.: Necropsy, Serology and Molecular Study Out of a Belgian Experience

**DOI:** 10.3390/pathogens9060413

**Published:** 2020-05-26

**Authors:** Fabien Grégoire, Raïssa Bakinahe, Thierry Petitjean, Samira Boarbi, Laurent Delooz, David Fretin, Marc Saulmont, Marcella Mori

**Affiliations:** 1 Association Régionale de Santé et d’Identification Animales (ARSIA), B-5590 Ciney, Belgium; fabien.gregoire@arsia.be (F.G.); thierry.petitjean@arsia.be (T.P.); laurent.delooz@arsia.be (L.D.); marc.saulmont@arsia.be (M.S.); 2 Bacterial Zoonoses of Animals Unit, Veterinary Bacteriology, Sciensano, 1180 Brussels, Belgium; Raissa.BakinaheNtamukunzi@sciensano.be (R.B.); Samira.Boarbi@sciensano.be (S.B.); David.Fretin@sciensano.be (D.F.)

**Keywords:** *Leptospira* spp., cattle, abortion, pathology, non-maintenance serovars, MAT, PCR, *lfb1*-phylogeny

## Abstract

Bovine leptospirosis is a bacterial zoonotic disease caused by pathogenic *Leptospira* spp. The pathology and epidemiology of this infection are influenced by the numerous existing serovars and their adaptation to specific hosts. Infections by host-maintained serovars such as Hardjo are well documented, unlike those from the incidental ones. In July 2014, an emerging phenomenon of an increased incidence of icteric abortions associated with leptospiral infection occurred in southern Belgium. First-line serological analyses targeting cattle-adapted serovars failed at initial diagnosis. This study provides a comprehensive description of laboratory findings—at the level of necropsy, serology and molecular diagnosis—regarding icteric and non-icteric abortions (n = 116) recorded during this time (years 2014–2015) and associated with incidental infection by serovars such as Grippotyphosa, Australis and Icterohaemorrhagiae. Based on these tests, a diagnostic pathway is proposed for these types of infection in cattle to establish an affordable but accurate diagnosis in the future. These investigations add insights into the understanding of the pathogenesis of bovine leptospirosis associated with serovars classically described as non-maintenance.

## 1. Introduction

Bovine leptospirosis is a bacterial zoonotic disease caused by pathogenic *Leptospira* spp., bacteria classified in about 300 serovars and 64 genomospecies, of which 37 belong to the pathogenic clade [1,2,3]. The pathology and epidemiology of this bacterial disease are influenced by the existing serovars and their adaptation to specific hosts in wildlife or livestock [1]. Depending on the serovar–host relationship, incidental and host-maintained infections are described. 

Leptospiral infections in cattle are known as incidental when involving non-maintenance pathogenic *Leptospira* spp. such as the serogroups Icterohaemorrhagiae, Hebdomadis, Grippotyphosa and Canicola [4,5,6]. These infections are transmitted through indirect or, more rarely, direct contact with an infected host, generally a wildlife reservoir (i.e., rodents for the serogroup Icterohaemorrhagiae [7,8]). On the other hand, cattle can act as the principal reservoir of the bacteria for the adapted *L. borgepetersenii* serovar Hardjobovis and *L. interrogans* serovar Hardjoprajitno. In this latter case, the transmission is more efficient and mainly occurs through direct contact with the contaminated urine or body fluids (milk or placental fluids) of infected animals residing in the same herd [9]. 

Infection by non-maintenance pathogenic *Leptospira* spp. is characterized by an acute form of the disease such as a high fever, hemolytic anemia, jaundice and multi-systemic illness [10]. Acute manifestation is rarely described for maintenance strains such as *L. borgepetersenii* serovar Hardjobovis and *L. interrogans* serovar Hardjoprajitno [11,12,13,14]. In this case, the host presents a sub-acute clinical phase of the disease that often goes unobserved. Bovine-adapted serovars follow a chronic course of infection that is associated with a prolonged renal carrier state of the adult animal associated with chronic renal diseases [15,16,17]. Chronically infected animals can excrete leptospires through urine, leading to increased environmental infection pressure. Economic issues can be important since these infections also cause reproductive failure, abortions, stillbirths, weak offspring and reduced milk production. Hence, it is a concern for public and animal health management. The management and diagnosis of infections due to *L. borgepetersenii* serovar Hardjobovis and *L. interrogans* serovar Hardjoprajitno in cattle are supported by numerous experimental and epidemiological studies [18,19,20,21,22,23,24]. *L. borgepetersenii* serovar Hardjobovis and *L. interrogans* serovar Hardjoprajitno have been shown to be causes of reproductive losses worldwide [25,26,27]. Instead, knowledge of the disease caused by non-maintenance serovars, other than from serological surveillance, is poor and linked to anecdotal reports [28,29,30]. The pathogenesis of reproductive disease due to *Leptospira* spp. remains poorly understood as well. Transplacental infection, occurring during the very limited time of maternal bacteremia, is supposed to be the sole cause of abortion [31]. 

In July 2014, an emerging phenomenon associated with an increased incidence of icteric abortions occurred in the southern region of Belgium [32]. Initial laboratory findings highlighted in dams serum high-level antibody titers against *Leptospira* serogroups Grippotyphosa and Australis, whilst a first-line ELISA-based serologic survey for *L. borgepetersenii* serovar Hardjobovis failed to identify the causative agent [32]. A definitive laboratory diagnosis was finally achieved at the animal national reference leptospirosis laboratory in the fall of 2014 by systematically associating positive serology in dams with the detection of pathogenic *Leptospira* spp. DNA in various organs of the abortuses. The conclusions were promptly reported to the national competent authorities (official national internal report). One epidemiological study thereafter conducted on prospective samples confirmed serological reactivity against *Leptospira* serogroups Grippotyphosa and Australis and provided genotypic indication in one sample of the same year, 2014 [33].

This study provides a comprehensive description of laboratory findings— at the level of necropsy, serology and molecular characterization—regarding the icteric and non-icteric abortions recorded during the years 2014–2015 and associated with leptospiral infection. Based on these tests and their relative intrinsic qualities, the goal of this work is to provide comprehensive tools for the clinical and laboratory diagnosis of bovine leptospirosis due to non-maintenance serovars. Besides, a diagnostic pathway is proposed to establish an affordable but accurate diagnosis of these cases in the future. Finally, these investigations add insights into the understanding of the pathogenesis and clinical manifestation of bovine leptospirosis associated with serovars classically described as non-maintenance.

## 2. Results

### 2.1. Characteristics at Necropsy of MAT Positive Abortions

A total of 116 fetuses were selected for the entire study. The gestational age varied from 3 to 9 months, with the majority of abortions occurring during the last trimester of gestation (Table 1). When considering the lesions assessed during necropsy, four entries were initially selected due to previously being observed in icteric abortions [32]: icterus (n = 52), splenomegaly (n = 59), coppery liver (n = 40) and peri-renal hemorrhage (n = 59). However, these entries accounted only partially for the microscopic agglutination test (MAT)-positive results observed (Table 2 and Table S1). Therefore, the presence of generalized hemorrhagic edema (n = 6) was additionally included to better understand the complete lesion panel associated with *Leptospira* infection. For each of these lesions, the association with a positive MAT result (regardless of the reactive serogroup) was evaluated. Four different seropositivity cut-offs for MAT were considered: 1/10, 1/100, 1/300 and 1/1000 (Table 2). A significant association was observed between seropositivity and the presence of icterus, splenomegaly or coppery liver for all of the considered cut-offs. A similar observation was made for the combination of these three lesions. By contrast, no link was established for the peri-renal hemorrhages, regardless of the MAT cut-off. An extended hemorrhagic pattern was associated with a positive MAT result for the cut-off of 1/1000 and, interestingly, observed only in the non-icteric abortion group (Table 2). 

### 2.2. Serological Pattern of Abortions

Serological analyses were performed on 88 individual dams’ sera, on 20 coupled dams’ sera and fetuses’ pleural fluids, and six individual fetuses’ pleural fluids. Pleural fluids were chosen as an alternative to missing post-mortem fetal sera because they reflect the serum antibody levels arising through passive diffusion [34]. These analyses allowed the obtaining of MAT profiles for all but two abortuses. Globally, a higher positivity rate was observed in abortuses with icteric (42/50, 80.7%) vs. non-icteric (33/64, 51.5%) patterns (Table S1). When both dam’s serum and fetus’ pleural fluid were tested and a positive serological MAT result was observed (18/20 abortions), positivity was observed only in the dam’s serum in 17 out of 18 cases (94.4%) (Table S1). For one single fetus, serologically positive reactions were observed in both the dam (high titer) and the pleural fluid of the fetus (low titer), with perfect agreement on the presumptive serogroup. The presumptive infecting *Leptospira* serogroup was defined in 65/75 (86.7%) of the MAT-positive abortuses, with the following frequencies: Grippotyphosa (n = 42), Australis (n = 13), Ballum (n = 4), Icterohaemorrhagiae (n = 2), Autumnalis (n = 1), Bataviae (n = 1), Pyrogenes (n = 1) and Tarassovi (n = 1). None of these serogroups belongs to the pathogenic *Leptospira* serovars classically described as maintenance in cattle. Cross-reactivities between the serogroups were observed, in order of decreasing frequency, between the serogroups Grippotyphosa and Ballum (18/75), Grippotyphosa and Autumnalis (14/75), Grippotyphosa and Australis (10/75), and Australis and Autumnalis (9/75) (Table S2).

### 2.3. Bacteriological Characteristics of Abortions

PCR was performed on 68 fetuses in available organs. A fetus was considered positive when a positive PCR result was reported in at least one organ. Again, clear high positivity rates were observed in abortuses with icteric (30/41, 73.17%) vs. non-icteric (1/27, 3.70%) patterns, as shown in Table 3. When associations were calculated between each lesion and a positive PCR result in abortuses, a significant link was only found for icterus, splenomegaly, coppery liver and the combination of these three lesions. No significant link was observed for peri-renal hemorrhages and general hemorrhagic pattern (Table 3). Based on the PCR results in the different organs, the placenta, spleen and liver appeared as the most appropriate of a fetus’ organs in which to look for non-maintenance *Leptospira* spp. colonization (Table 4). The positive rate was significantly higher in the placenta than in the kidney (χ^2^ = 6.15, *p*-value = 0.013). The median Ct (cycle threshold) values were averagely high in all the organs, and most of the Cts were above 30. For all but five of the tested fetuses (68), the MAT results for the dam’s serum were available to define the possible relations between PCR and MAT tests (Table S3). Cohen’s kappa coefficient, as well as the relative sensitivity and specificity towards PCR, were calculated for each cut-off of the MAT test. The cut-off of 1/300 showed the highest concordance value (kappa = 0.679, substantial agreement), with a relative sensitivity and a specificity approaching 85% (Table S3).

### 2.4. Molecular Typing of Leptospira spp.

**Figure 1:**
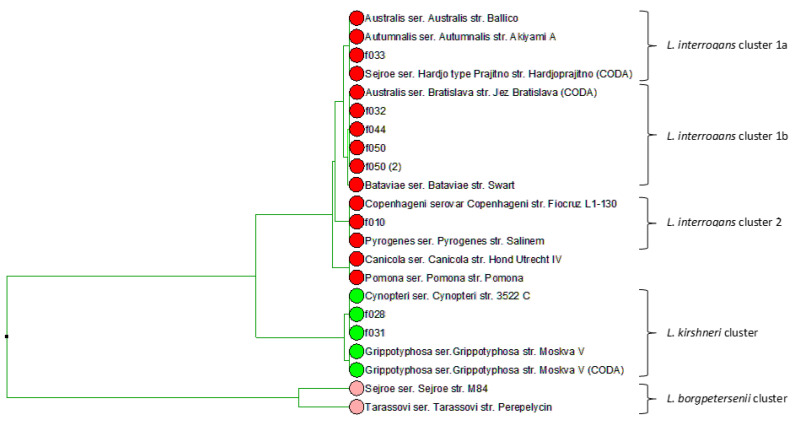
*lbf1*-derived phylogeny of Leptospira-induced icteric abortions and reference strains. Red, green and rose circles indicate strains of *L. interrogans*, *L.kirschneri* and *L. borgepetersenii* species, respectively. Reference strains are listed with their serogroup, serovar and strain name. Abortuses are indicated with their fetus ID as in Table S1.

To better understand the epidemiology of *Leptospira*-induced abortions in this study and to identify, at the species level, the *Leptospira* infecting strain, the analysis of the sequence polymorphism in the fibronectin-binding protein gene (*lfb1*) gene was performed by using both high-resolution melting analysis (HRMA) and amplicon sequencing. The melting curve analysis of eight fetuses indicated the presence of two main types of PCR amplicon, with different melting temperatures (Tm), highlighting the presence of at least two *Leptospira* species causing abortions. The first cluster, with an average 81.8 °C Tm, was assigned, by comparison with the Tm of reference strains, to the *Leptospira interrogans* group; the second cluster, with a one-degree difference in Tm (average of 82.8 °C) was assigned to the *Leptospira kirschneri* group (Figure S1). Phylogenetic analyses of the *lfb1* sequence in seven fetuses and reference strains (ours or those available in databases) corroborated observations by HRMA. The results, supported by the amplification of a minimum of 238 bp of sequence (accession numbers KY373222 to KY373229), evidenced the heterogeneity of the species involved in abortions: three *Leptospira interrogans* clusters (1a, 1b and 2) including the serovars Australis Ballico, Autumnalis and Hardjoprajitno (cluster 1a); the serovars Australis Bratislava and Bataviae (cluster 1b); the serovars Pyrogenes and Copenhageni (cluster 2); one *Leptospira kirschneri* cluster including the reference sequences of the serovars Cynopteri and Grippotyphosa (Figure 1). Except for the fetus f044, where the presumptive serogroup could not be identified by MAT, the *Leptospira* genospecies based on molecular typing is compatible with the presumptive serogroups determined by MAT in dams (Table 5).

## 3. Discussion

Abortion is a well-known manifestation of leptospirosis in cattle. Nevertheless, extended anatomo-pathological descriptions of fetuses infected with leptospires are rarely available, especially for serovars other than *L. borgepetersenii* serovar Hardjobovis. The emergence of abortions due to non-maintenance leptospires observed in 2014 in southern Belgium showed that general icterus was the most striking lesion reported in the abortuses [32]. Jaundice is an acute manifestation commonly reported in newborn calves infected by *Leptospira* spp., due to hemolysis following hemolysin production. This lesion was also reported in fetuses in cases of experimental abortions with *L. borgepetersenii* serovar Hardjobovis [35]. On the basis of the abortuses included in this study, coppery liver and splenomegaly, besides icterus, were significantly associated with a positive result for *Leptospira*, for both serology and bacteriology. These lesions in abortuses are not pathognomonic, and their appreciation depends on the state of conservation of the cadaver. Less distinct lesions such light icterus could be missed by an unaware or untrained eye. Despite these minor drawbacks, necropsy of the fetuses remains, as strongly confirmed in this study, an efficient tool in the global context of abortion surveillance. As of the Belgian experience, such monitoring proved its efficacy during the re-emergence episodes of the Schmallenberg virus and, to a lesser extent, brucellosis [36,37]. In addition to the three mentioned lesions, generalized hemorrhagic edema has been observed in some abortuses and was shown to be associated with high antibody titers in dams. No specific serovar was associated with this lesion. Petechial and ecchymotic hemorrhages on internal organs completed the picture in most of these cases, whereas jaundice was absent in these fetuses except in one. The high *Leptospira* titers associated with generalized hemorrhagic edema suggested a per-acute phenomenon in this case. This feature might correspond to the first stage of the disease leading to an icteric pattern or even represent a completely different pathogenesis of bovine leptospirosis. Indeed, the outcome of *Leptospira* infection varies depending on the infecting serovars but also on host specificities or host–pathogen interaction [38].

Globally, serological analyses indicated that non-maintenance *Leptospira* spp. serogroups were, in different proportions, the causes of the icteric-hemorrhagic abortions observed in this study, namely serogroups Grippotyphosa, Australis, Ballum, Icterohaemorrhagiae, Autumnalis, Bataviae, Pyrogenes and Tarassovi. The seropositive results could not be attributed to vaccination, as a vaccine for cattle is not marketed in Belgium. The comparison of our results with European trends is difficult, although our data are in line with the marked increase in leptospirosis infection observed in the year 2014 [39,40]. However, a complete epidemiological picture of *Leptospira* spp. serogroups in cattle across Europe is unavailable. The reasons for this are various; seroprevalence studies are often restricted to a specific timeframe, data are not centralized at the European level for animals and the standardization of the MAT analyses is subject to constraints. The Grippotyphosa, Australis, Icterohaemorrhagiae and—to a lesser extent—Autumnalis and Bataviae serogroups have been observed in clinically relevant cattle herds in France [41]. Although this in line with our data, information is missing to ascertain whether the reproductive disorders described in France were similar to the ones observed in our study. Seroprevalence studies performed in cattle herds in Italy show that the non-maintenance serogroups Australis and Icterohaemorrhagiae are observed at high titer levels, together with the maintenance serovars Hardjo and Pomona, while Ballum, Canicola, Grippotyphosa and Tarassovi are rare and at minimal titers [42]. In Spain, the relevant cattle serovars are Bratislava, Hardjo and Pomona [43,44]. Limited seroprevalence data are present in Belgium to understand the relationship between the serogroups found in this study and their possible presence/spread in other (reservoir) animals; Australis and Grippotyphosa are the main serogroups found in dogs [45], and they were also detected in muskrats [34,46]. In humans, a marked increase in autochthonous cases was reported in 2014 [47], as observed in other parts of Europe [39], but a univocal association of the serovars involved in bovine icteric abortions in 2014 with human cases could not be established (National Reference Laboratory personal communication).

Molecular diagnosis, supported by serology, highlighted two mains genospecies responsible for abortions in this study: *L. interrogans* and *L. kirschneri*. Although this was relatively clear from our previous investigations [32], here, we demonstrated by *lfb1* phylogeny that infections could be sourced back to genetically diverse strains (for instance, at least three clusters for the *L. interrogans* species) and not to a unique “outbreak” clone. This could probably explain the heterogenous serological response against *Leptospira* agglutinins and variation observed with the MAT analyses. Although the *L. interrogans* cluster included sequences that were very closely related to the *L. interrogans* serovar Hardjoprajitno, the involvement of this serovar was excluded in our conclusion as it was not supported by serological analyses operated both by ELISA [32] and MAT.

Together with the above observations allowing a better understanding of the *Leptospira* pathogenesis in cattle, this study provided additional insights for the laboratory diagnosis of bovine leptospirosis. First, antibodies against *Leptospira* spp. were detected by MAT in the dam’s serum and not in the pleural fluid of the fetuses, despite positive PCR results for fetus organs. This indicated that the dam’s serum was the most appropriate sample type for serodiagnosis in this study. Although we previously demonstrated that pleural fluids, in absence of post-mortem sera, are informative for leptospiral infective status [34], these samples taken from the bovine fetus are possibly not relevant in case of cattle infection with leptospires. It can also be speculated that we could not identify seropositive reactions because the fetuses were in bacteremia, which is associated in humans with an absent or very low serological antibody response [48]. Second, the cut-off for MAT was lowered to as low as 1/10 dilution. In classical serological diagnosis, the MAT cut-off is kept at 1/100 (or at 1/400 for higher specificity); the OIE manual suggests lowering this threshold in the case of serosurveillance studies [49]. We suggest lowering this threshold also in case of clustered cases recorded in a defined time-lapse and linked to unusual phenomena. Third, cross-reactivities between serovars were likely to occur, and, with the exception of few cases, they did not hamper the final main serogroup presumptive identification, which was in high agreement with the genospecies attribution. Fourth, leptospiral antigens in abortuses were found in additional organs other than the placenta, kidneys, liver and adrenal glands [50]. As suggested by our study, *Leptospira* spp. are likely to be also detected by PCR in the spleen. This is not surprising since this organ acts like a blood filter and thus is susceptible to sequestering bacteria. Last, this study strongly supports, in the case of poorly loaded DNA samples, the use of *lfb1* phylogeny with the HRMA or sequencing methodology for outbreak-clustered case investigations to provide enough discriminatory genetic power for difficult samples [51,52].

Some limitations could be present in our study. A positive PCR result in the abortus indicates that the bacteria reached the gravid uterus, suggesting a strong link between the presence of *Leptospira* and abortion. However, most of the Ct results from PCR were high, suggesting a low quantity of bacteria in fetal tissues, and some cases could have be missed due to the limit of detection of the method. In addition, in our study, a negative PCR result could not allow the exclusion of *Leptospira* from the abortion causes because of the non-systematic testing of all the organs. For example, the placenta was not systematically transmitted with the fetus for analysis. Additionally, a defect in the collection of the lesional data during necropsy cannot be excluded, especially at the beginning of the emergence of leptospiral abortions (01/09/14) when the pathologists were less aware of the suspicious lesions. One last limitation is related to the number of cases upon which this study is based to determine the associations between lesions and MAT results. This was mainly due to the epidemic course, with a drop in the icteric cases in October 2014.

**Figure 2:**
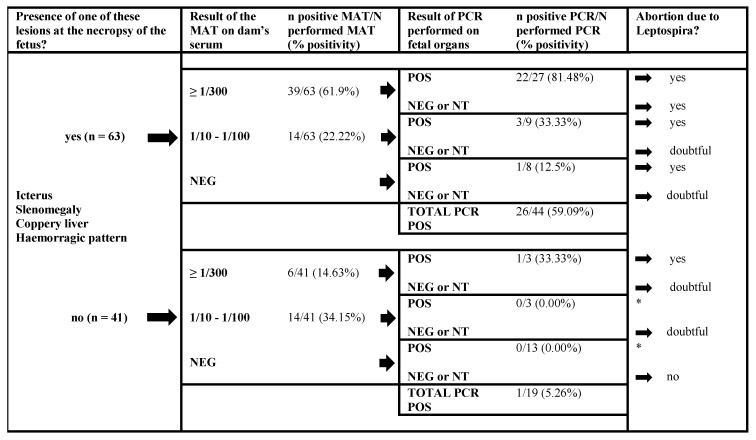
Recommended diagnostic approach for abortuses according to the results of necropsy, MAT on a dam’s serum and PCR on fetal organs performed in series, for a conclusion about *Leptospira* spp. as the cause of the abortion. NT: non tested, * did not occur in this study.

Based on overall our findings, an algorithm is proposed to diagnose, in first-line settings, leptospiral infections in cattle in an accurate and economically affordable manner (Figure 2). This approach is particularly reliable in contexts where no vaccination strategies in cattle are in place. In the case of a fetus showing suspicious lesions, MAT would be an initial preferred choice. In two thirds of the cases, it gave a positive result above 1/300 that can likely assign the abortion to an infection of the dam by *Leptospira* spp. PCR on fetal organs was not as efficient for attesting *Leptospira* as a causal agent; only about one quarter of the fetuses showed a positive result. However, using PCR in parallel to MAT allows the confirmation of more cases and the targeting of samples on which culture or molecular typing can be done. In the case of a doubtful diagnosis, complementary tests should be considered, i.e., a PCR on a dam’s vaginal discharge/urine [53,54] or a paired sampling of the dam’s serum to ascertain an increase in the serological titer [49]. This laboratory approach—knowing the history of vaccination, reproduction and clinical symptoms in the dams—enables the accurate etiological diagnosis of bovine leptospirosis caused by non-maintenance *Leptospira* spp.

## 4. Materials and Methods

### 4.1. Study Protocol

The bovine abortuses selected for this study (116) were declared in southern Belgium (Walloon Region) between 27 August 2014 and 15 June 2015 and sampled within the framework of a national surveillance program of abortions in cattle. The study period relates to a time where unusual manifestations of congenital jaundice cases in bovine fetuses were firstly observed in Belgium [30]. In order to have equal representations of icteric and non-icteric abortuses, non-icteric cases were randomly selected among those declared in the same timeframe. The herds where these abortions occurred were not vaccinated as no *Leptospira* vaccine has marketing authorization in Belgium.

### 4.2. Necropsy and Sample Collection

All abortions were subjected to a standardized necropsy. The thoracic, abdominal and pelvic cavities were examined, as were the brain, the content of the abomasum and—if present—the placental cotyledons. Any lesion was systematically recorded, including (1) icterus, corresponding to a yellow coloring of the connective tissues, especially the visceral adipose ones; (2) splenomegaly, defined as an enlargement of the spleen with a muddy consistency of the organ; (3) coppery liver, the yellow coloration of the hepatic parenchyma with the absence of the modification of the liver volume or its consistency; (4) peri-renal hemorrhages, bleeding under the renal capsule; and (5) generalized hemorrhagic edema (subcutaneous and cavitary) associated, in some cases, with multifocal hemorrhages at the surfaces of organs. The estimation of the gestational ages of the abortuses was made based on the fetal length and external characteristics [55]. Pleural fluid from abortuses (n = 26) was sampled during necropsy, and serum from the dams (n = 108) was collected around the time of the abortion. Samples were stored at 4 °C or at −20 °C for subsequent processing if the tests could not be performed in the immediate days after necropsy.

### 4.3. Strain and Culture Conditions

*Leptospira* strains used for the MAT serological test were maintained in liquid Ellinghausen-McCullough-Johnson-Harris (EMJH) medium supplemented with 0.2% w/v yeast extract and 10% fetal calf serum (PAA Laboratories GmbH). The cultures were grown at 29 °C and inoculated weekly by 1:50 dilution. Strains were quality controlled twice-yearly against a panel of hyperimmune positive serovar-specific sera (AMC, Amsterdam).

### 4.4. MAT

MAT was performed on fetal pleural fluids and the dams’ sera as recommended by the OIE manual [49]. The *Leptospira* strains used in the MAT analysis of this study included pathogenic isolates belonging to twelve different serogroups as previously described [34]. Briefly, the sera were diluted in PBS and incubated with live strains at the desired threshold dilution in single well of a 96-well plate. The agglutination reaction was achieved following a 1 h incubation at 37 °C. A loopful of the reaction mix was placed on a slide, and a reading acquired under an upright microscope at 20x magnification (Olympus, Japan). Serum was considered positive if it presented agglutination in at least one of the tested serogroups. Endpoint titers were determined by starting from an initial dilution of 1:10 and using a three-fold dilution until the last condition showing 50% agglutination. The presumptive serogroup was identified by taking the highest agglutination titer of the serum with one particular pathogenic *Leptospira* strain.

### 4.5. DNA Extraction and Diagnostic Real Time PCR

For nucleic acid extraction, available tissue samples (spleen, placenta, kidneys, adrenal glands, liver, lung, brain or hepatic lymph nodes) were macerated in physiological water, and 200 µL of this homogenate was mixed with 235 µL of lysis-binding solution (MagMax, Applied Biosystem, Paisley, UK) and supplemented with lysozyme at a final concentration of 1 mg/mL. After incubation for one hour at 37 °C, samples were processed for extraction as defined by the manufacturer (MagMax nucleic acid extraction kit, Applied Biosystem). Alternatively, about 20 mg of the roughly chiseled organ was mixed with 180 μL of solution NM1 (LSI MagVet™ Universal Isolation Kit, Thermo Fisher Scientific, Paisley, UK) and, after 1 min of agitation, incubated for 30 min at 70 °C +/− 5 °C. The lysate was then agitated and centrifuged for 1 min at 6000 g. The DNA purification was performed with an automat KingFisher Flex 96^TM^ according to manufacturer’s instructions. The diagnostic PCR, targeting the *Lipl32*gene, was performed with in house primers [56] or with the LSI TaqVet^TM^ PathoLept^TM^ (Thermo Fisher Scientific, Merelbeke, BE) as defined by the manufacturer. For in house settings, the reaction mix consisted of 2x absolute mix (Applied Biosystem), 300 µM each of forward and reverse primers and 100 µM of the TaqMan probe. The thermal conditions were those defined elsewhere [56]. The PCR reactions were performed in triplicate on an ABI7500 thermocycler (Thermo Fisher Scientific). Samples showing amplification in at least one PCR reaction (Ct < 45) were considered as positive.

### 4.6. High-Resolution Melting Analysis

The molecular typing of positive diagnostic PCR samples was based on the polymorphism of the fibronectin-binding protein gene (*lfb1*) [51,52]. This method has been proven as reliable to distinguish genomic *Leptospira* species and to investigate outbreaks, because it does not require samples to have as high bacterial loads as other methods. Polymorphism analysis was achieved with both high-resolution melting analysis (HRMA) and, if PCR amplicons were sufficiently loaded, by *lfb1* gene fragment sequencing. PCR amplifications were achieved using primers previously described [51,52] on a Light Cycler 480 II (Roche) using the LightCycler FastStart DNA Master SYBR Green I (Roche Applied Science). The cycling conditions included a holding step at 95 °C for 10 min; a real-time run of 95 °C for 8 s, 60 °C for 5 s, and 72 °C for 12 s for 45 cycles; and a melting step of 30 °C (from 65 °C to 95 °C) with an acquisition rate of 5 °C. Melting curve analyses were performed with the Light Cycler 480 software release 1.5.1.62 by using default settings.

### 4.7. Sequencing

PCR amplicons were collected and sent to GENEWIZ (United Kingdom) for outsourcing the sequencing. The subcontracted service included PCR amplicon purification and sequencing by the dideoxy chain-termination procedure. The quality of the chromatograms was analyzed with the Bionumerics software package V 6.6 (Applied Maths, Belgium). An assembly between forward and reverse sequences was obtained with default settings. The sequences obtained in this study have been deposited in GenBank (accession numbers KY373222 to KY373229). Multiple alignments were done, setting the gap penalty at 0%, and cluster analysis was performed using the unweighted-pair group method with an arithmetic mean algorithm (UPGMA).

### 4.8. Statistical Analysis

Associations between necropsy findings and MAT/PCR results were evaluated with the Chi-square test, or Fisher’s exact test in cases of any expected frequency inferior to 5 (significance level of *p* < 0.05). The statistical tests were performed with SigmaPlot 11.0 (Systat Software, San Jose, CA). In addition, for the analysis of the lesional data, odds ratios were calculated with 95% confidence intervals (95% CI). For the agreement between the diagnostic tests (MAT and PCR), Cohen’s kappa statistic with 95% confidence intervals was calculated. The interpretation for the kappa statistic was as follows: <0.2, slight agreement; 0.2–0.4, fair agreement; 0.4–0.6, moderate agreement; 0.6–0.8, substantial agreement; and >0.8, almost perfect agreement [57]. The kappa coefficients were calculated using WIN EPISCOPE 2.0 [58]. In addition to the kappa coefficients, the relative sensitivities and specificities of the MAT towards PCR were calculated with 95% "exact" Clopper–Pearson confidence intervals.

## Figures and Tables

**Table 1:** Distribution of the autopsied fetuses according to their gestational age, presence of icterus (other lesions not included) and positivity rates of MAT (cut-off of 1/10).

Month of Abortion	N Autopsied	Icteric (N Positive/ N Analyzed for Lesions)	Positive MAT in Dam’s Sera (N Positive/N Tested)	Positive MAT in Pleural Fluids (N Positive/N Tested)	Positive PCR (N Positive/N Tested)
<5	8	0/8	5/7	0/1	0/0
5	2	0/2	1/2	0/0	0/1
6	5	0/5	4/5	0/0	0/2
7	7	2/6	4/7	0/0	0/5
8	43	18/40	30/42	1/9	13/25
9	51	32/50	31/45	0/16	19/37
**TOTAL**	**116**	**52/111**	**75/108**	**1/26**	**32/70**

**Table 2:** Association between necropsy findings in bovine abortuses and results of MAT (positive reaction for any serogroup) performed on sera of the corresponding dams. P = presence; A = absence.

Lesion		N Fetuses	Positivity Cut-Off of MAT							
			1/10		1/100		1/300		1/1000	
			% Positive MAT (n)	OR (95% IC) χ^2^ and *p*-Value	% Positive MAT (n)	OR (95% IC) χ^2^ and *p*-Value	% Positive MAT (n)	OR (95% IC) χ^2^ and *p*-Value	% Positive MAT (n)	OR (95% IC) χ^2^ and *p*-Value
**Icterus**	**P**	44	90.91 (40)	12.61 (3.94–40.40) χ^2^ = 20.99 *p* < 0.001	88.64 (39)	13.55 (4.56–40.24) χ^2^ = 24,91 *p* < 0.001	77.27 (34)	14.28 (5.33–38.29) χ^2^ = 30,04 *p* < 0.001	43.18 (19)	7.14 (4.09-12.48) χ^2^ = 12.59 *p* < 0.001
	**A**	52	44.23 (23)		36.54 (19)		19.23 (10)		9.62 (5)	
**Splenomegaly**	**P**	54	83.33 (45)	6.67 (2.60–17.09) χ^2^ = 15.41 *p* < 0.001	77.78 (42)	5.69 (2.33–13.91) χ^2^ = 13,94 *p* < 0.001	61.11 (33)	4.43 (1.84–10.67) χ^2^ = 10.24 *p* = 0.001	33.33 (18)	3.00 (1.12–8.04) χ^2^ = 3.61 *p* = 0.057
	**A**	42	42.86 (18)		38.10 (16)		26.19 (11)		14.29 (6)	
**Coppery liver**	**P**	36	86.11 (31)	5.43 (1.86–15.85) χ^2^ = 9.31 *p* = 0.002	80.56 (29)	4.43 (1.68–11.66) χ^2^ = 8.47 *p* = 0.004	69.44 (25)	5.99 (2.35–15.29) χ^2^ = 13.64 *p* < 0.001	44.44 (16)	5.20 (2.83–9.56) χ^2^ = 10.01 *p* = 0.002
	**A**	60	53.33 (32)		48.33 (29)		31.67 (19)		13.33 (8)	
**Icterus + splenomegaly + coppery liver**	**P**	29	89.66 (26)	7.03 (1.94–25.49) χ^2^ = 9.16 *p* = 0.002	86.21 (25)	6.44 (2.02–20.52) χ^2^ = 10.06 *p* = 0.002	75.86 (22)	6.43 (2.38–17.33) χ^2^ = 13.41 *p* < 0.001	44.83 (13)	4.14 (2.46–6.97) χ^2^ = 7.26 *p* = 0.007
	**A**	67	55.22 (37)		49.25 (33)		32.84 (22)		13.33 (11)	
**Peri-renal hemorrhages**	**P**	54	64.81 (35)	0.92 (0.39–2.16) χ^2^ = 0.0007 *p* = 0.978	57.41 (31)	0.75 (0.33–1.72) χ^2^ = 0.22 *p* = 0.636	44.44 (24)	0.88 (0.39–1.98) χ^2^ = 0.01 *p* = 0.918	25.93 (14)	1.12 (0.44–2.85) χ^2^ = 0.007 *p* = 0.934
	**A**	42	66.67 (28)		64.29 (27)		47.62 (20)		23.81 (10)	
**Extended hemorrhagic pattern**	**P**	6	83.33 (5)	2.76 (0.31–24.65) *p* = 0.661	83.33 (5)	3.49 (0.39–31.12) *p* = 0.397	83.33 (5)	6.54 (0.73–58.26) *p* = 0.09	66.67 (4)	7.00 (1.19–41.04) *p* = 0.033
	**A**	90	64.44 (58)		58.89 (53)		43.33 (39)		22.22 (20)	
**TOTAL**		96	65.63 (63)		60.42 (58)		45.83 (44)		25.00 (24)	

**Table 3:** Association between necropsy findings in bovine abortuses and PCR results for the diagnosis of leptospirosis. P = presence; A = absence.

Lesions		N Fetuses	% Positive PCR (n)	OR (95% IC) χ^2^ and *p*-Value
**Icterus**	**P**	41	73.17 (30)	70.91 (8.57–586.89) χ^2^= 31.67 (*p* < 0.001)
	**A**	27	3.70 (1)	
**Splenomegaly**	**P**	45	64.44 (29)	19.03 (3.95–91.81) χ^2^= 16.89 (*p* < 0.001)
	**A**	23	8.70 (2)	
**Coppery liver**	**P**	31	70.97 (22)	7.60 (2.58–22.38) χ^2^= 12.97 (*p* < 0.001)
	**A**	37	24.32 (9)	
**Icterus + splenomegaly + coppery liver**	**P**	28	75.00 (21)	9.00 (2.95–27.45) χ^2^= 14.65 (*p* < 0.001)
	**A**	40	25.00 (10)	
**Peri-renal hemorrhages**	**P**	49	46.94 (23)	1.22 (0.42–3.54) χ^2^= 0.008 (*p* =0.930)
	**A**	19	42.11 (8)	
**Extended hemorrhagic pattern**	**P**	3	33.33 (1)	0.58 (0.05-6.76) *p* = 1
	**A**	65	46.15 (30)	
**TOTAL**		**68**	**45.59 (31)**	

**Table 4:** PCR results obtained in various organs of the autopsied fetuses^*^ (n = 68).

Organ	N Positive/N Tested	Positivity (%)	Median Ct Value	Range Min–Max	
Spleen	18/45	40.00	36.67	31.06	41.45
Placenta ^a^	17/32	53.13	34.14	27.52	41.41
Kidney ^a^	4/21	19.05	33.92	28.10	38.64
Adrenal glands	1/15	6.67	36.60	36.60	36.60
Liver	5/14	35.71	34.58	31.90	37.60
Lung	2/10	20.00	34.61	32.30	36.92
Brain	0/5	0.00	/	/	/
Hepatic lymph nodes	2/3	66.67	37.45	36.70	38.20

* One abortus might be positive for several organs. ^a^ significantly different (*p* = 0.013).

**Table 5:** Comparison of results in autopsied fetuses according to high-resolution melting analysis (HRMA), *lfb1* phylogeny and serology in dams.

		HRMA rtPCR		*lfb1* Sequencing	Serology of Dam	Necropsy
Fœtus ID	Sample ID	Tm	Species Group Attribution	Species Group Attribution	MAT	Group
f050	15095924-1509927-159915	81.92	*interrogans*	*interrogans* cluster 1b	1/500 Australis	Icteric
f033	14040522.1-1610225.2	81.93	*interrogans*	*interrogans* cluster 1a	1/1000 Icterohaemorrhagiae	Icteric
f010	14048419.1-1610225.3	81.69	*interrogans*	*interrogans* cluster 2	1/500 Australis	Icteric
f032	14045546	81.71	*interrogans*	*interrogans* cluster 1b	-	Icteric
f044	14057925-1417554.1-1417819	81.72	*interrogans*	*interrogans* cluster 1b	ND (1/100 Ballum 1/100 Grippothyphosa)	Icteric
f028	15050216-1506066.2	82.96	*kirschneri*	*kirschneri* cluster	1/100 Grippotyphosa	Icteric
f007	14047394.1-1610225.1	82.82	*kirschneri*	-	1/500 Grippotyphosa	Icteric
f031	14044550	82.76	*kirschneri*	*kirschneri* cluster	-	Icteric

## Supplementary Materials

The following are available online at https://www.mdpi.com/2076-0817/9/6/413/s1, Table S1. Detailed results of necropsy, the MAT and the real-time PCR performed on 116 abortuses for the diagnosis of leptospirosis. SPL: splenomegaly; CL: coppery liver; PRH: peri-renal hemorrhages; SD: serum of the dam; PLF: pleural fluid. Dilution*: agglutination less than 50%. Table S2: Cross-reactions between serovars of *Leptospira* spp. observed on 75 dams’ sera submitted to MAT (cut-off of 1/10). Table S3: Comparison of results of PCR performed on fetuses and results of MAT performed on the serum of the corresponding dams. Figure S1: Melting curves derived from analysis of the *lfb1* sequence polymorphisms in *Leptospira* spp. icteric abortions by HRMA.
