# Supplementary material for: Laboratory Diagnosis of Bovine Abortions Caused by Non-Maintenance Pathogenic Leptospira spp.: Necropsy, Serology and Molecular Study Out of a Belgian Experience
Source: Pathogens. 2020 May 26;9(6):413. doi: 10.3390/pathogens9060413 (PMC7350382; doi:10.3390/pathogens9060413)
Supplement: Supplementary file 1 [file pathogens-09-00413-s001.zip › supplementary/Figure S1.pdf]

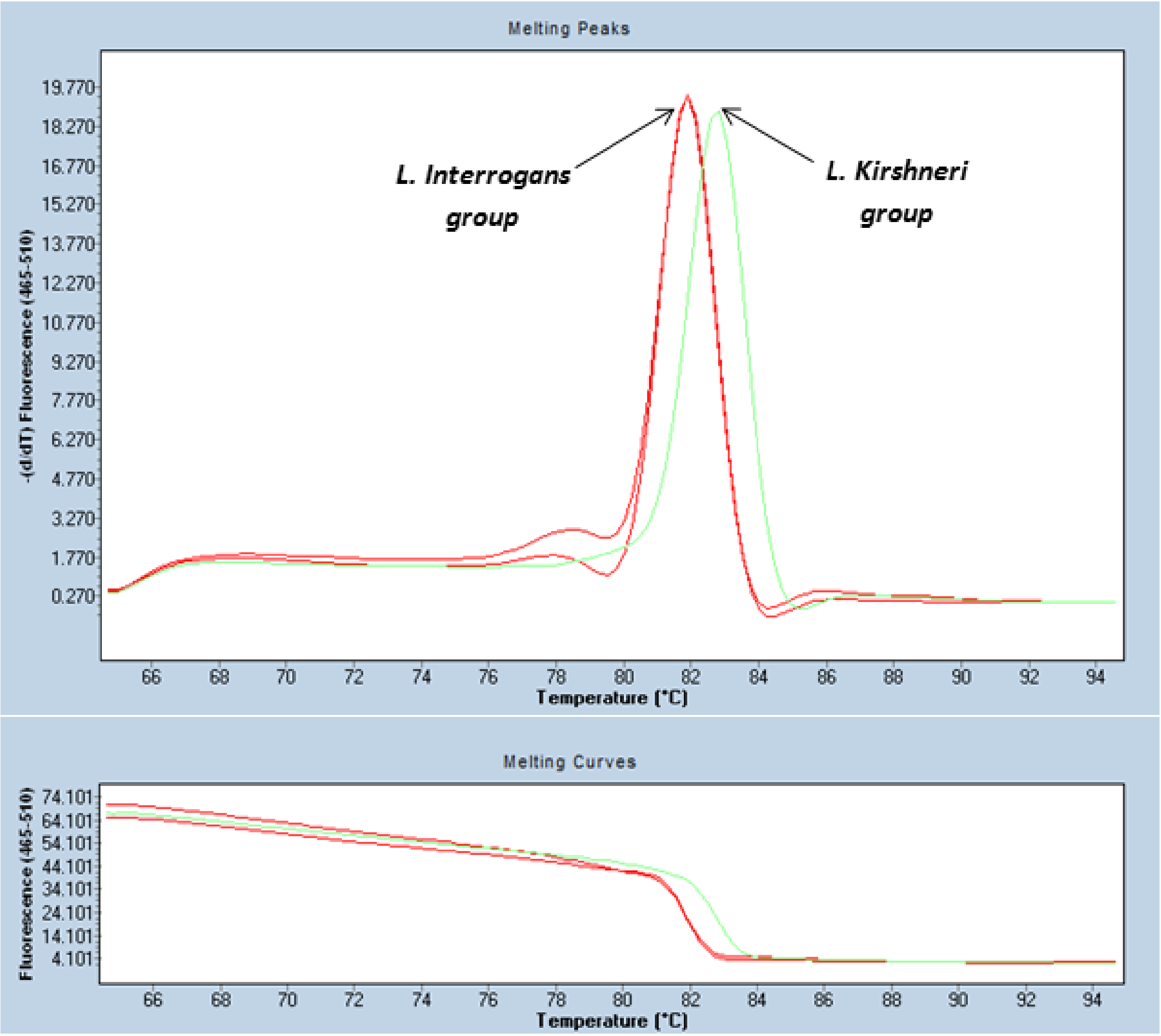

Figure S1. Melting curves derived from analysis of the *lfb1* sequence polymorphisms in *Leptospira* spp. icteric abortions by HRMA. Derivative (upper panel-melting peaks) and raw fluorescent (lower panel-melting curves) emission data recorded during the melting step. Two main profiles were observed with different Tms, the *L.interrogans* group (red lines) and the *L.kirschneri* group (green lines).
