# Supplementary material for: Laboratory Diagnosis of Bovine Abortions Caused by Non-Maintenance Pathogenic Leptospira spp.: Necropsy, Serology and Molecular Study Out of a Belgian Experience
Source: Pathogens. 2020 May 26;9(6):413. doi: 10.3390/pathogens9060413 (PMC7350382; doi:10.3390/pathogens9060413)
Supplement: Supplementary file 1 [file pathogens-09-00413-s001.zip › supplementary/Table S3.pdf]

Table S3. Comparison of the results of the PCR performed on fetuses and the results of microscopic agglutination test performed on the serum of the corresponding dams for the diagnosis of leptospirosis.

	N fetuses	Positivity cut-off of MAT			
		**1/10**	**1/100**	**1/300**	**1/1000**
		**n positive MAT**	**n positive MAT**	**n positive MAT**	**n positive MAT**
**PCR positive**	27	26	26	23	14
**PCR negative**	36	14	13	6	3
**TOTAL**	63	40	39	29	17
**Ser (95% CI)**		96.30 (81.03-99.91)	96.30 (81.03-99.91)	85.19 (66.27-95.81)	51.86 (31.95-71.33)
**Spr (95% CI)**		61.11 (43.46-76.86)	63.89 (46.22-79.18)	83.33 (67.19-96.63)	91.67 (77.53-98.25)
**Kappa (95% CI)**		0.54 (0.31-0.77)	0.57 (0.34-0.80)	0.68 (0.43-0.92)	0.46 (0.22-0.69)
