# Supplementary material for: Laboratory Diagnosis of Bovine Abortions Caused by Non-Maintenance Pathogenic Leptospira spp.: Necropsy, Serology and Molecular Study Out of a Belgian Experience
Source: Pathogens. 2020 May 26;9(6):413. doi: 10.3390/pathogens9060413 (PMC7350382; doi:10.3390/pathogens9060413)
Supplement: Supplementary file 1 [file Table S2.pdf › supplementary/Table S2.pdf]

Table S2. Cross-reactions between serovars of *Leptospira* spp. observed on 75 dams' sera submitted to MAT (cut-off of 1/10).

Frequencies	Grippotyphosa	Canicola	Pomona	Ballum	Ictero	Javanica	Australis	Autumnalis	Bataviae	Pyrogenes	Tarassovi	Sejroe
**Grippotyphosa**		0	2	18	6	/	10	14	1	3	1	1
**Canicola**			0	1	1	/	0	0	0	1	0	0
**Pomona**				1	2	/	2	3	1	1	0	0
**Ballum**					4	/	7	6	0	5	1	1
**Ictero**						/	1	2	1	2	0	0
**Javanica**						/	/	/	/	/	/	/
**Australis**								9	1	3	2	0
**Autumnalis**									1	1	1	0
**Bataviae**										0	0	0
**Pyrogenes**											2	0
**Tarassovi**												0
**Sejroe**												
